# Establishment of orthotopic osteosarcoma animal models in immunocompetent rats through muti-rounds of in-vivo selection

**DOI:** 10.1186/s12885-024-12361-z

**Published:** 2024-06-07

**Authors:** Mengyu Yao, Zehua Lei, Feng Peng, Donghui Wang, Mei Li, Guoqing Zhong, Hongwei Shao, Jielong Zhou, Chang Du, Yu Zhang

**Affiliations:** 1 Department of Orthopedics, Guangdong Provincial People’s Hospital (Guangdong Academy of Medical Sciences), Southern Medical University, Guangzhou, 510080 China; 2 GuangDong Engineering Technology Research Center of Functional Repair of Bone Defects and Biomaterials, Guangzhou, 510080 China; 3 Hebei Key Laboratory of Biomaterials and Smart Theranostics, School of Health Sciences and Biomedical Engineering, Hebei University of Technology, Tianjin, 300130 China; 4 Department of Biomedical Engineering, School of Materials Science and Engineering, South China University of Technology, Guangzhou, 510006 China

**Keywords:** Animal model, Osteosarcoma, Orthotopic transplantation, Cell line, Immune system

## Abstract

Immunodeficient murine models are usually used as the preclinical models of osteosarcoma. Such models do not effectively simulate the process of tumorigenesis and metastasis. Establishing a suitable animal model for understanding the mechanism of osteosarcoma and the clinical translation is indispensable. The UMR-106 cell suspension was injected into the marrow cavity of Balb/C nude mice. Tumor masses were harvested from nude mice and sectioned. The tumor fragments were transplanted into the marrow cavities of SD rats immunosuppressed with cyclosporine A. Through muti-rounds selection in SD rats, we constructed orthotopic osteosarcoma animal models using rats with intact immune systems. The primary tumor cells were cultured in-vitro to obtain the immune-tolerant cell line. VX2 tumor fragments were transplanted into the distal femur and parosteal radius of New Zealand white rabbit to construct orthotopic osteosarcoma animal models in rabbits. The rate of tumor formation in SD rats (P1 generation) was 30%. After four rounds of selection and six rounds of acclimatization in SD rats with intact immune systems, we obtained immune-tolerant cell lines and established the orthotopic osteosarcoma model of the distal femur in SD rats. Micro-CT images confirmed tumor-driven osteolysis and the bone destruction process. Moreover, the orthotopic model was also established in New Zealand white rabbits by implanting VX2 tumor fragments into rabbit radii and femurs. We constructed orthotopic osteosarcoma animal models in rats with intact immune systems through muti-rounds in-vivo selection and the rabbit osteosarcoma model.

## Introduction

Osteosarcoma, the most common primary malignant tumor of the bone, is often occurs in adolescents. Osteosarcoma primarily occurs in the metaphyses of long bones, including the distal femur, proximal tibia, and humerus [1, 2]. The five-year survival rate of all the patients at all the stages of osteosarcoma is approximately 60-70% [3]. In particular, osteosarcoma patients with lung metastasis maintain even poorer prognoses. Although neoadjuvant chemotherapy has enhanced the management of osteosarcoma by facilitating preoperative planning and potentially modifying postoperative treatment, the prognosis and five-year survival rate of osteosarcoma patients have not markedly improved over the past few decades [4]. One of the main factors that hinder the development of osteosarcoma treatment is the lack of a full understanding of the cellular and molecular mechanisms underlying the development of this condition. A suitable animal model can help in promoting the investigation of cellular and molecular mechanisms related to osteosarcoma [5].

The current in-vivo osteosarcoma models include spontaneous, inducible, and transplantation osteosarcoma models and other genetically engineered models [6]. Among them, transplantation models, including homografts and xenografts, are the most important ones. Xenografts have been developed by implanting human osteosarcoma cells or tissues into immunodeficient murine hosts, such as athymic nude mice, which possess a greater tumor-forming efficiency and can be manipulated and evaluated easily [7]. In 1993, the first successful xenogeneic tumor model was constricted by Berlin. He developed a spontaneous metastasis model in athymic mice by utilizing the v-Ki-ras-oncogene-transformed human osteosarcoma cell line (KRIB), which was orthotopically implanted into the tibial bones of nude mice [8]. Despite the numerous advantages of xenogeneic models, the loss of immune system in immunodeficient murine models does not allow proper tumor-host interactions and hinders the investigation of tumor initiation and metastasis and the function of immune cells in antitumor immune reactions or tumor immune evasion in the tumor environment [9–11]. In contrast, homografts of osteosarcoma (including orthotopically transplanted homografts and heterotopically transplanted homografts) have several advantages in biologically relevant host microenvironments and are currently the most extensively used method for the establishment of osteosarcoma tumor models. Heterotopically transplanted models can be established in the subcutis or musculature. For example, Chen et al. used the subcutaneous model of Balb/C mice to study the in-vivo photothermal antitumor effects of three-dimensional (3D), printed Wesselsite [SrCuSi_4_O_10_] nanosheets integrated with polycaprolactone (SC/PCL) composite scaffolds [12]. However, without the participation of normal bone stromal cells and matrix proteins and cytokines, the subcutaneous and intramuscular models cannot simulate critical host-tumor cell interactions. In orthotopically transplanted models, tumor cells or tissues introduced into the intramedullary cavity of either the proximal tibia or distal femurs or implanted adjacent to the bone periosteum properly emulate the complex interactions between tumor cells and the intercellular matrix and the bone microenvironment from where the tumor originated [7, 13]. Mice are commonly used to establish orthotopically transplanted models. For example, Liao et al. implanted K7M2 tumor masses in sites near the tibias of Balb/C mice to evaluate the bone repair function of methacrylated gelatin/methacrylated chondroitin sulfate hydrogel hybrid gold nanorods and nanohydroxyapatite [14]. However, the bones of mice are small and are only suitable for the study of nanomaterials and hydrogel materials; they cannot meet the clinical needs associated with bone-implanted materials. Development of a method to construct an osteosarcoma model in rats with a intact immune system remains a challenge.

In this study, we constructed an in-situ osteosarcoma model of the distal femur in Sprague–Dawley (SD) rats using a stepwise selection strategy. The tumorigenic rate, tumorigenic time, and the tumor growth behavior were investigated systematically. Furthermore, this method was used to construct an orthotopic osteosarcoma model in New Zealand white rabbits.

## Materials and methods

### Cell and tissue cultures

The rat osteosarcoma cell line UMR-106 and the VX2 tumor tissue were used in this study. These were purchased from Shanghai Zhong Qiao Xin Zhou Biotechnology Co., Ltd., and Tongpai Shanghai Biotechnology Co., Ltd, respectively. UMR-106 cells were grown and maintained in Dulbecco’s modified Eagle’s medium (DMEM) containing 4500 mg/liter glucose (DMEM-HG, Gibco, USA), supplemented with 10% fetal bovine serum (FBS, Gibco, USA), 100 U/ml penicillin, and 100 µg/ml streptomycin. The cells were maintained at 37 °C in 5% CO_2_.

### Animals

The athymic Balb/C nude mice (male, 5 weeks old) and SD rats (male, 8 weeks old) were purchased from the Hunan SJA Laboratory Animal Co., Ltd. The New Zealand white rabbits used in this study were obtained from the Animal Experimental Center of South Medical University, China. All animals were kept in laminar flow cabinets under specific, pathogen-free (SPF) conditions. All manipulations on animals were conducted according to the ARRIVE guidelines for animal experiments, using an approved protocol from the Laboratory Animal Research Center of South China University of Technology.

### Construction of orthotopic osteosarcoma model of the distal femur in SD rats

#### Expansion of rat osteosarcoma UMR-106 cells

**Fig. 1:**
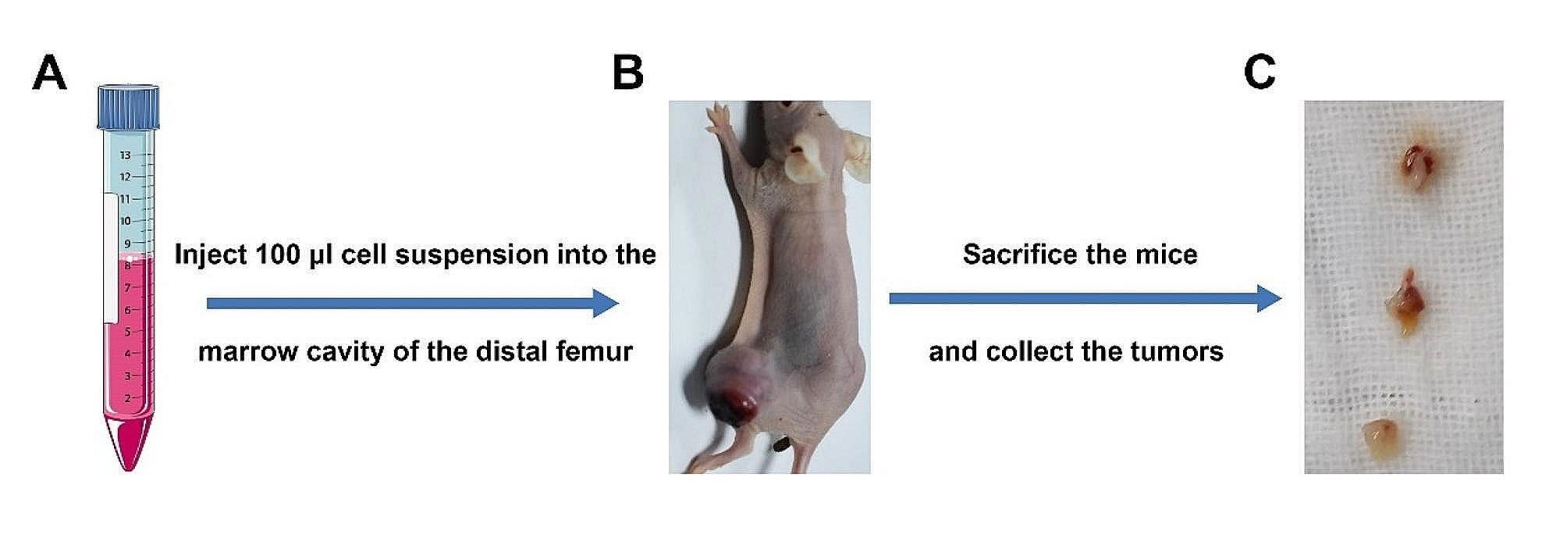
Resuscitation and expansion of rat osteosarcoma UMR-106 cells. (**A**) UMR-106 cells suspended at a density of 1 × 10^7^ /ml. (**B**) Tumor formed in the distal femur after 10–14 days. (**C**) Tumor mass was sectioned into tissue volumes of 2 × 2 × 2 mm³

The rat osteosarcoma UMR-106 cells were harvested with trypsin-ethylenediaminetetraacetic acid and resuspended in a solution of FBS-free DMEM and Matrigel (volume ratio = 1:1) at a density of 1 × 10^7^ /ml. In total, six Balb/C nude mice were anesthetized with intraperitoneal (i.p.) injection of 1.5% sodium pentobarbital at a ventral position. One side of the skin of the hindlimb was sterilized using iodine. After exposure of the trochlear groove, the needle of a 10 ml syringe was used to puncture the groove to reach the marrow cavity in a direction parallel to the longitudinal axis of the femur, and the needle was routed to expand the cavity. Subsequently, approximately 1 × 10^6^ UMR-106 cells were slowly injected into the marrow cavity. When the cell suspension gelled, the needle was pulled out, the skin was closed immediately, and pressure was applied to stop the bleeding. The tumor size of the distal femur was measured with a caliper, and the tumor volume was calculated. When the diameter of the tumor reached 10 mm (about 10 days), the mice were sacrificed, and the tumors were collected and minced into tissue volumes of 2 × 2 × 2 mm³; these were labeled as samples at generation P0. Some tumors were collected for in-vitro cultures to obtain the UMR-106-P0 cell line. The process is shown in Fig. 1.

### Replantation of orthotopic osteosarcoma of the distal femur in SD rats

110 SD rats were used for this study. Three days before replantation, the rats were immunosuppressed with cyclosporine A (10 mg/kg). After shaving, their hind limbs were locally disinfected with iodine and cut at lengths of 0.5 cm along the lateral side of the patella. After separating the muscles and fascia tissues, the lateral femoral condyle was exposed and drilled with an electric drill to create a hole 3 mm in diameter and 4 mm in depth. The tumor fragment of generation P0 of the UMR-106 cells was placed inside the hole. Thereafter, the skin was sutured and re-disinfected. After the surgery, all rats were immunosuppressed with i.p. injections of cyclosporine A (10 mg/kg) for one week and intramuscular (i.m.) injections of penicillin consecutively for three days (200,000 U/mouse) to prevent infection. The rats receive tumor tissue injections on both femurs. All rats were observed for wound healing and tumor formation. The percentage of tumor formation was evaluated using the following equation:

1 $${\rm{Tumor}}\,{\rm{ formation }}\,{\rm{ratio}}\,{\rm{ = }}\,{\rm{An/At }}\,{\rm{ \times }}\,{\rm{ 100\% }}$$

where *An* is the number of tumors formed, and *At* is the total number of tumor transplantation sites.

The tumor mass collected from the first 10 SD rats was identified as the P1 generation.

### In-vivo selection and establishment of immune-tolerant cell lines

In this study, we used an in-vivo stepwise selection strategy to select immune-tolerant cell lines. 10 rats were used for in-vivo selection for each generation. When the P1 generation developed (tumor size was 2–3 cm in diameter), the tumors were collected and sectioned into 2 × 2 × 2 mm³ tumor fragments and implanted into the marrow cavities of the distal femurs of new recipient SD rats, which were earlier immunosuppressed with cyclosporine A. The rats were then closely observed for tumor progression. The tumors formed in the immunosuppressive recipient rats were marked as generation P2 and isolated for re-implantation into the marrow cavities of immunosuppressed SD rats to form a new generation. These operations were repeated. The dosage of cyclosporine A is reduced by 2 mg/kg per round until it is not injected at P6.

**Fig. 2:**
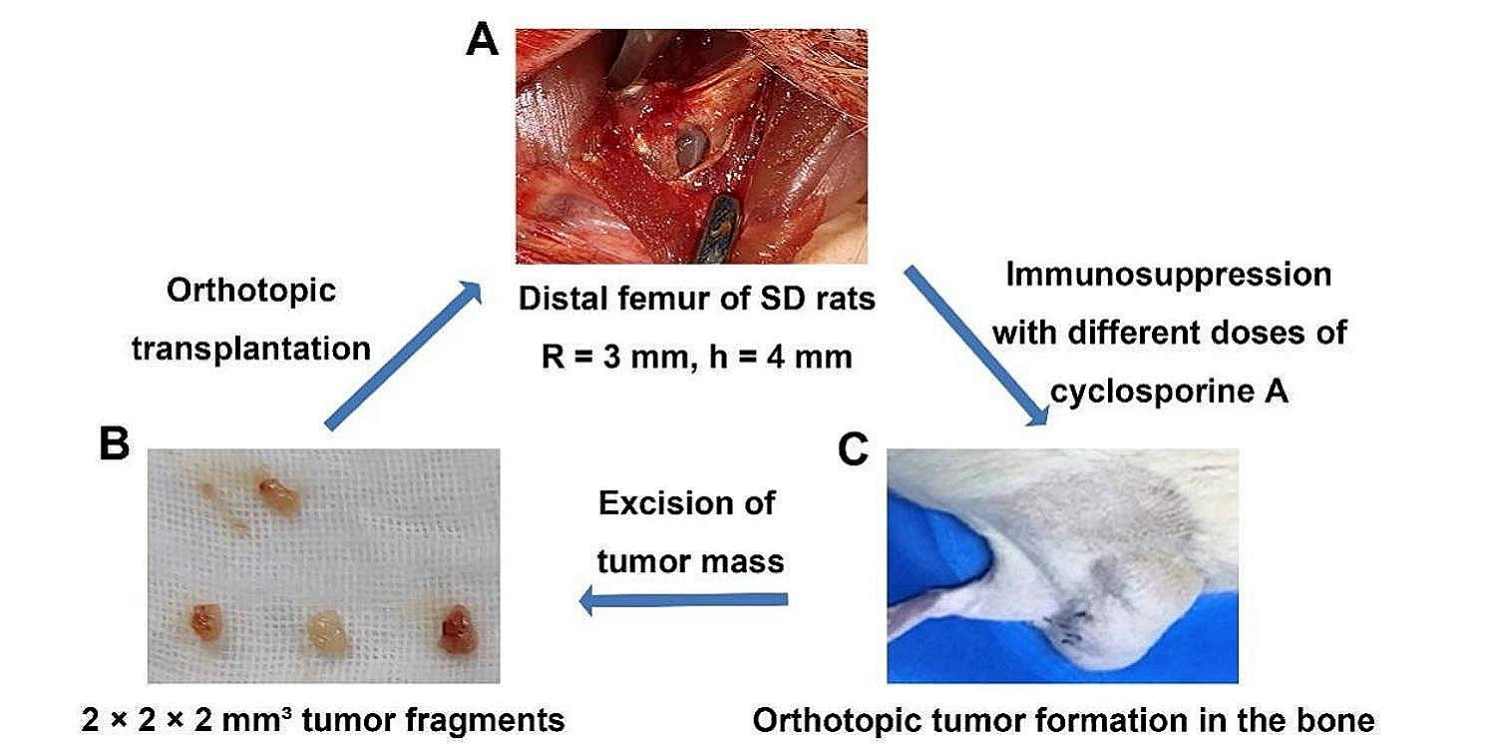
Process of immune domestication. (**A**) Bone defects were observed in the distal femurs of SD rats. (**B**) Orthotopic tumor mass was sectioned into 2 × 2 × 2 mm³ tumor fragments. (**C**) Orthotopic tumor formation in the bone

Thereafter, new tumors formed in the SD rats with intact immune system were isolated and re-implanted into the marrow cavities of new recipients SD rats with intact immune systems. The same procedure was repeated. We scanned the rats with micro-computer tomography (CT) until the rate of tumor formation in SD rats became 100%, and then isolated the bones and tumor masses for histological analyses. Furthermore, we initiated the in-vitro culturing of primary tumor cells to obtain the immune-tolerant cell line UMR-106-11. The experimental process is shown in Fig. 2.

### Establishment of in-situ osteosarcoma model of the distal femur in SD rats with intact immune systems

We implanted the tumor mass of UMR-106-P11 into the marrow cavities of distal femurs of SD rats subjected to the same operation as detailed above to establish the in-situ osteosarcoma model of the distal femur in SD rats with intact immune systems.

## Construction of in-situ osteosarcoma model in New Zealand white rabbits

### Expansion of rabbit VX2 tumors

Two healthy male New Zealand white rabbits, weighing 2–2.5 kg, were used in this study. Each rabbit was anesthetized with 3% sodium pentobarbital (30 mg/kg), which was injected in the ear vein. The skin of the right hip was disinfected with iodine, and an incision (length: 1 cm) was made longitudinally. After the subcutaneous fascia was separated and cut, the VX2 tumor tissue was implanted under it to expand. Thereafter, the layered suture closure and disinfection were conducted. The wound healing and growth of tumor were observed every day. When the subcutaneous tumor diameter developed to ∼2–3 cm (about 6 days), it was removed and sectioned into 3 × 3 × 3 mm³ tumor fragments for the subsequent operation.

### Transplantation of VX2 tumor of the distal femur in New Zealand white rabbits

Six male New Zealand white rabbits (weighing 2–2.5 kg) were anesthetized with 3% sodium pentobarbital (30 mg/kg) and injected in the ear vein. Both the hindlimbs and hips were disinfected. After sectioning the anterolateral intermuscular layer of the femur and separating the muscles, the distal femur (the border of trochlear groove of femur and bone cortex of frontal femur) in 2 cm range was exposed and drilled with an electric drill to form a bone defect (3 mm in diameter and deep into the inner bone cortex of the femur), which was closely followed by the transplantation of VX2 tumor fragments. Sterilized medical bone wax was used to close the defect. After the layer was disinfected, irrigated, and sutured, each rabbit was injected i.m. with penicillin (600,000 U/rabbit) to prevent infection. Rabbits were kept in standard facilities, and the status of wound healing and tumor formation was monitored (observations were conducted on a daily basis). Tumor formation was evaluated as mentioned above.

### Transplantation of VX2 tumor of parosteal radius in New Zealand white rabbits

Six male New Zealand white rabbits (weighing 2–2.5 kg) were anesthetized with 3% sodium pentobarbital (30 mg/kg) with injection in the ear vein. The front limb skin was disinfected and cut to expose the middle and lower parts of the radius, and the tumor fragments were transplanted into the parosteal radius. The deep fascia layer and skin were sutured to immobilize the tumor fragments. The rabbits were injected with penicillin (600,000 U/rabbit) i.m. to prevent infection. Animals were kept in standard facilities, and the status of wound healing and tumor formation was observed daily. The percentage of tumor formation was evaluated as mentioned before.

**Fig. 3:**
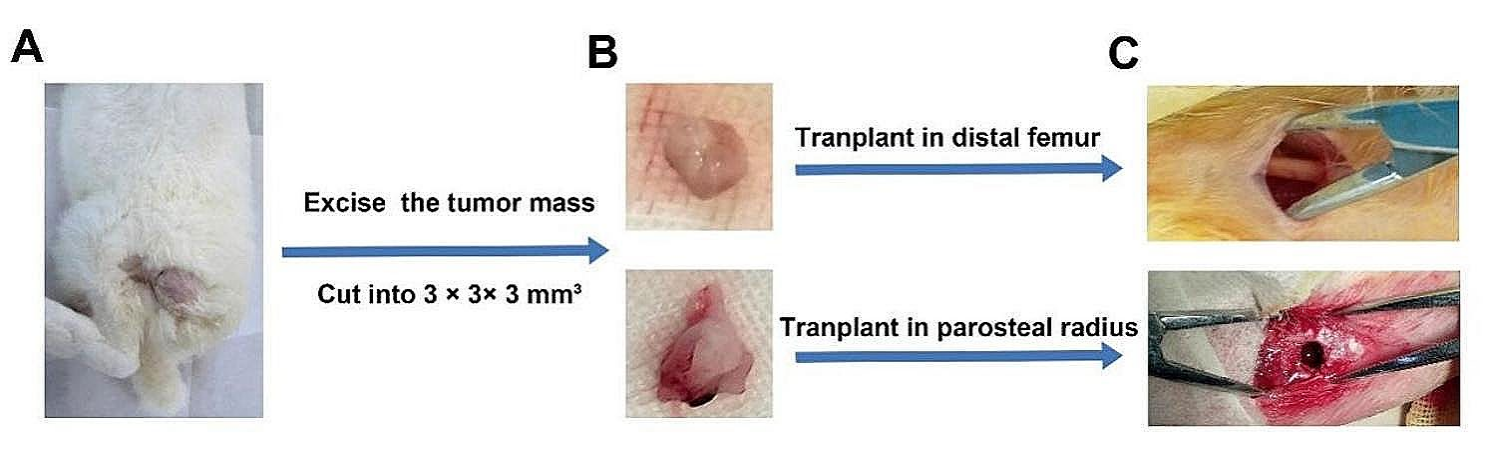
Construction of a femur VX2 tumor model orthotopic. (**A**) Subcutaneous VX2 tumor formation. (**B**) Subcutaneous tumor mass was sectioned into 3 × 3 × 3 mm³ tumor fragments. (**C**) Bone defects were made in the distal femur and parosteal radius

The process of construction of the in-situ osteosarcoma model of the distal femur in VX2 is shown in Fig. 3.

### Evaluation of tumor growth and metastasis formation

All animals were monitored every day to evaluate the local tumor growth. When the tumor developed to a 2–3 cm in diameter, the animals were anesthetized deeply and sacrificed to excise the bones with tumors and lungs to evaluate the metastasis.

### Primary cell culture and cell morphology

The tumors excised from the rats for primary culture were minced into tissue volumes of 1 mm³, and the surrounding soft tissue was removed. Tumor cells were isolated with a tumor dissociation kit (Miltenyi Biotec, Germany) and cultured in DMEM-HG (Gibco, USA) supplemented with 10% FBS (Gibco, USA). When the cells reached 70–80% confluency, the culture medium was removed. The cells were washed with PBS and cultured in fresh media. Cell morphology was observed and imaged with a light microscope (Eclipse 80i, Nikon, Japan).

### Histological examination and immunohistochemistry

Following the standard protocols, the tissues isolated from the body were fixed in 4% formalin, and the bones were decalcified. After embedding, the tissues were cut into 4 μm sections and stained with hematoxylin and eosin (H&E). Immunohistochemistry was performed using rabbit anti-Ki67 and rabbit anti-SATB2 antibodies. The tissue slides were blocked with 10% goat serum, followed by incubation with HRP-conjugated goat anti-rabbit as the secondary antibody.

### Micro-CT

Micro-CT images were obtained, using the ZKKS-MCT-Sharp-II micro-CT scanner (Zhongke Kaisheng, China), to estimate the extent of bone destruction. Animals were scanned after they were euthanized. The 3D image was reconstructed with the NRecon software (SkyScan, Bruker Corporation). Bone destruction was analyzed using the scan software.

## Results

*UMR106 rat osteosarcoma cell morphology and* in-situ *osteosarcoma of the distal femur in Balb/C nude mice*.

**Fig. 4:**
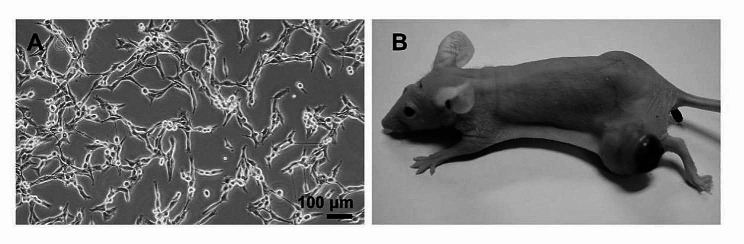
Morphological observation of the UMR-106 cell line and in-situ osteosarcoma of the distal femur in Balb/C nude mice. (**A**) Morphology of UMR-106 cells (100×). (**B**) Representative orthotopic tumor formation in distal femurs of Balb/C nude mice on day 14

As shown in Fig. 4A, UMR-106 grew in clumps. The cells presented an elongated and spindled morphology with active hyperplasia and frequent cell division. The cells cultured in DMEM-HG proliferated vigorously, and the cell density reached 90% in 24 h (passage at a split ratio of 1:3) or 48 h (passage at a split ratio of 1:6). UMR-106 cells were injected first in the marrow cavities of the distal femurs of Balb/C nude mice to harvest tumor fragments to increase the rate of tumor formation in SD rats. Localized swelling was detectable 3–4 days after intrabone marrow injections. At day 7, we observed the formation of localized small tumors. At day 14, all injection sites of the six studied mice formed tumor masses with diameters ranging from 8 to 12 mm (Fig. 4B).

### In vivo selection

**Fig. 5:**
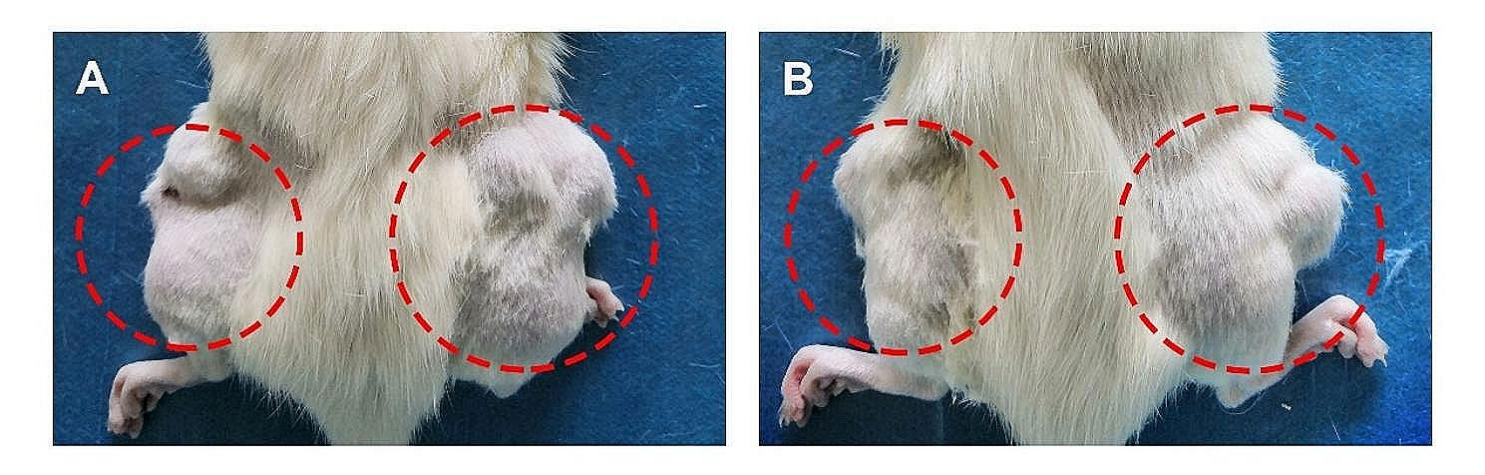
Orthotopic tumorigenesis of P11 tumor tissue in the distal femurs of Sprague–Dawley (SD) rats (indicated by the red dotted circles)

Tumor masses that grew in the distal femurs of Balb/C nude mice were collected, sectioned into 2 × 2 × 2 mm³ fragments, and then replanted into SD rat femurs. Notably, the rate of tumor formation in SD rats (P1 generation) pretreated with cyclosporine A was 30% (Table 1). Specifically, we selected an immune-tolerant cell line by gradually decreasing the dose of cyclosporine A, which was injected into SD rats in the subsequent generations. After four selection rounds, the UMR-106 tumor fragment could form tumor masses in SD rats (P5 generation) with intact immune systems without the use of cyclosporine A. The rate of tumor formation in generation P5 was 55%. Furthermore, to increase the rate of tumor formation in SD rats with intact immune systems, we conducted six rounds of consecutive in-vivo selections. Ultimately, we obtained useful results, as all the SD rats studied herein contained orthotopic tumors that resulted in pulmonary metastasis. Two typical results of orthotopic tumorigenesis of P11 tumor tissue in the distal femurs of SD rats are shown in Fig. 5.

**Table 1:** The number of tumors, tumor formation rate, and tumor formation time for each generation of rats (20 legs for each generation)

	Tumor Formation Number	Tumor Formation Ratio	Tumor Formation Time (days)
P1	6	30%	14
P2	7	35%	13
P3	9	45%	13
P4	9	45%	13
P5	11	55%	12
P6	13	65%	10
P7	15	75%	10
P8	15	75%	9
P9	17	85%	9
P10	17	85%	8
P11	20	100%	7

### Primary tumor formation and Micro-CT images

**Fig. 6:**
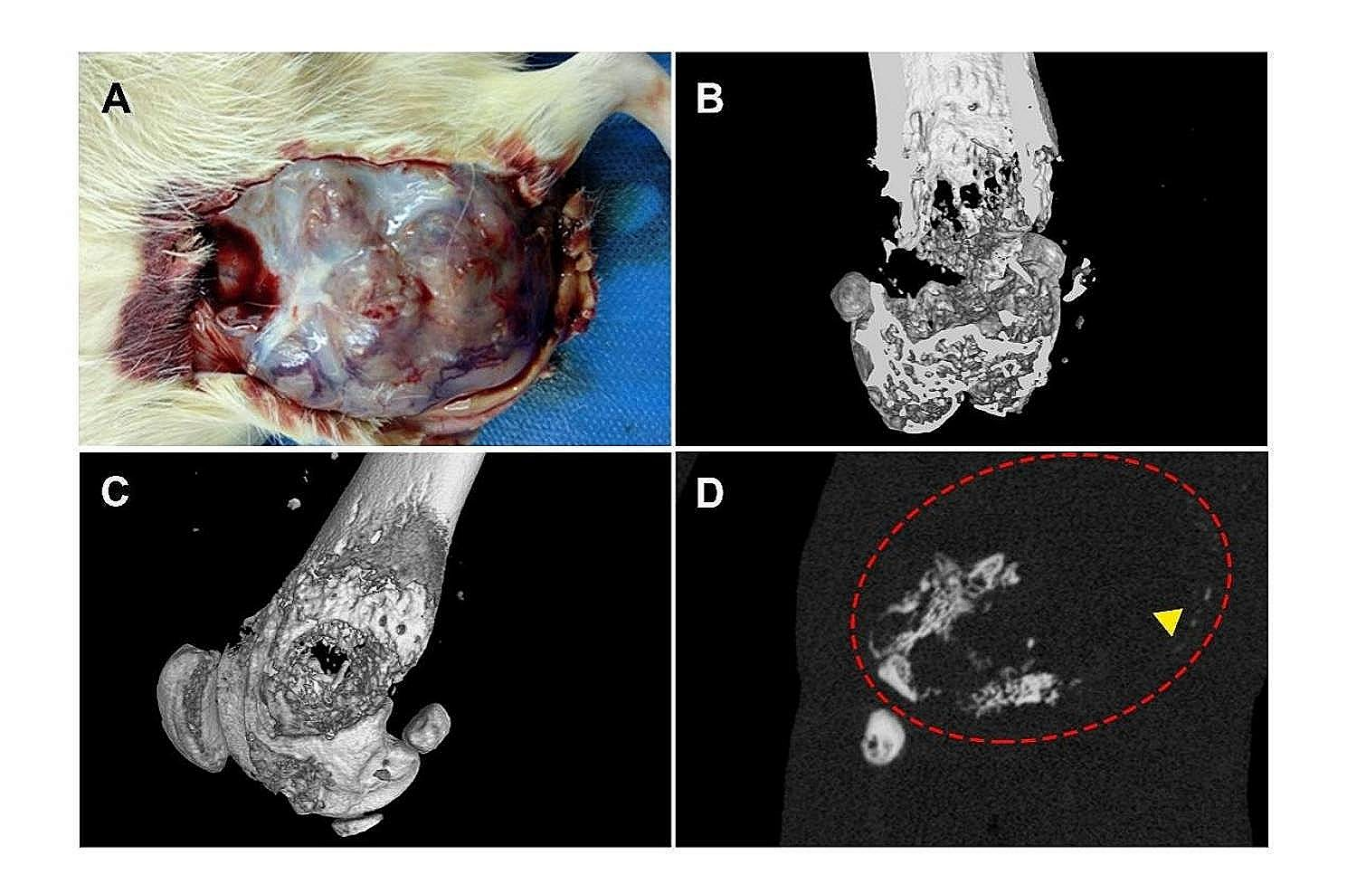
Tumor and micro-computer tomographic (CT) images of osteosarcoma in the distal femurs of SD rats orthotopic. (**A**) Massive solid tumor grew on the distal femur of an SD rat at generation P11. (**B, C**) Three-dimensional (3D) reconstruction of micro-CT scan from representative femur lesions. (**D**) Transverse images of the representative soft tissue mass

Autopsies in P11 generation rats identified excessively large solid tumors, which grew on the distal femur, and abundant blood vessels were observed on the surface of the tumor (Fig. 6A). A rich blood supply and active proliferation was consistent with the growth pattern of clinical osteosarcoma. Micro-CT images confirmed tumor-driven osteolysis and the bone destruction process (Fig. 6B and C), in conjunction with localized neoplastic osteogenic calcification (indicated by the yellow triangle in Fig. 6D).

### Pulmonary metastasis and H&E staining

**Fig. 7:**
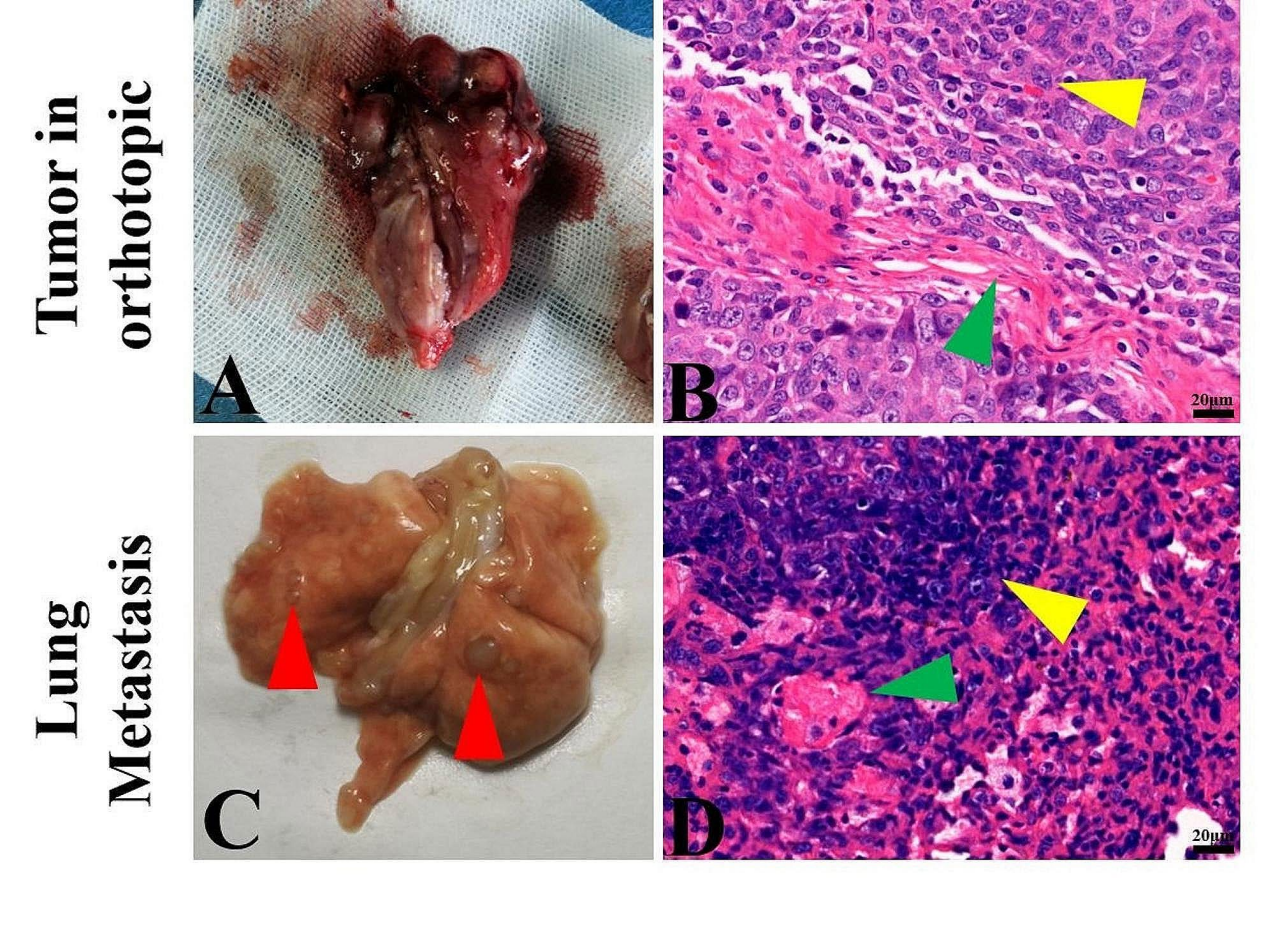
Tumor and hematoxylin and eosin (H&E) staining of osteosarcoma in the distal femur and lung metastasis in SD rats. (**A**) In-situ tumor in a rat at generation P11. (**B**) Histological sections of tumors orthotopic (yellow triangle: tumor tissue; blue triangle: neoplastic osteoid bone). (**C**) Massive metastatic nodules in the lungs (red triangle). (**D**) Histological sections of lung metastasis (yellow triangle: metastatic tumor tissue)

The tumor tissues were then separated and collected. Based on naked-eye observations, the tumor tissues presented white fish-flesh-like morphologies (Fig. 7A). Histological analyses revealed increased amounts of tumor cells surrounded by small amounts of fibrous connective tissues (Fig. 7B). In addition, it was obvious that many small metastasis nodules formed on the lungs (Fig. 7C). Furthermore, H&E staining confirmed that massive metastasis occurred in the lung parenchyma (Fig. 7D) that corresponded with the metastasis of the osteosarcoma.

### Primary culture

**Fig. 8:**
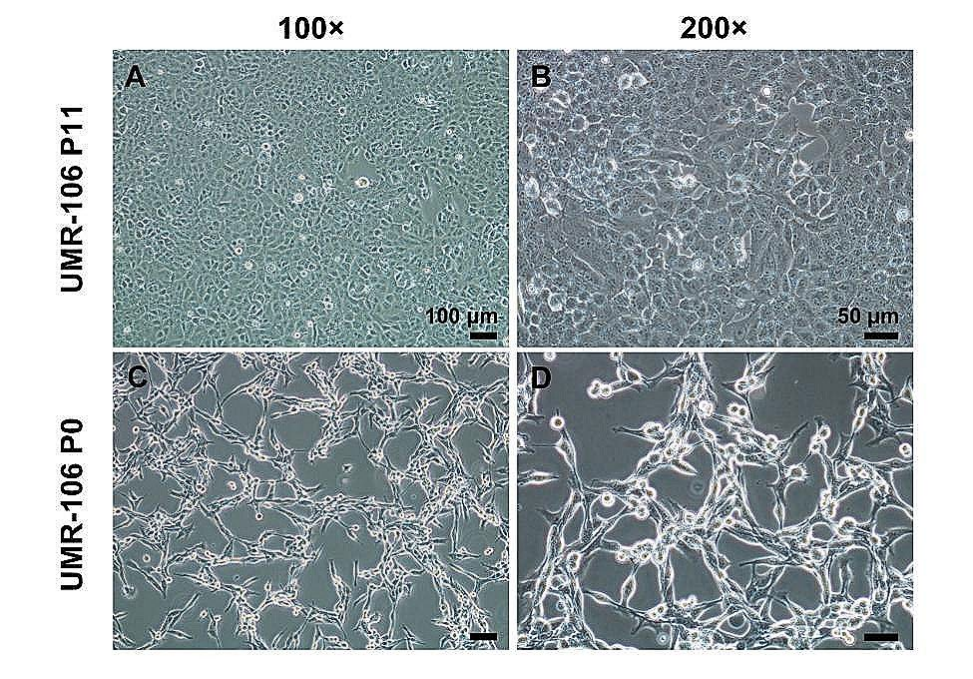
Morphological comparisons between the immune-tolerant UMR-106 cells (generation P11) and the primary UMR-106 cell line (generation P0). (**A, B**) UMR-106-P11 generation presented spindle-shaped morphology and a few protrusions among cells (100× and 200×). (**C, D**) UMR-106-P0 generation presented large oblate morphology and multiple protrusions (100× and 200×)

Tumor tissues in SD rats at P0 and P11 generations were isolated for primary culture to obtain immune-intolerant UMR-106 cells (labeled as UMR-106-P0) and immune-tolerant UMR-106 cells (labeled as UMR-106-P11), respectively. As shown in Fig. 7, both these cell lines contained many large nuclei. UMR-106-P11 presented a spindle shape and a few protrusions among cells (Fig. 8A and B). By contrast, UMR-106-P0 presented large oblate morphology and multiple protrusions (Fig. 8C and D).

### Construction of orthotopic osteosarcoma model of the distal femur in SD rats with intact immune systems

The cell suspension of UMR-106-P11 were injected into the marrow cavities of distal femurs of SD rats, and an orthotopic osteosarcoma model of the distal femur in SD rats with intact immune systems was successfully established (labeled as generation P12).

**Fig. 9:**
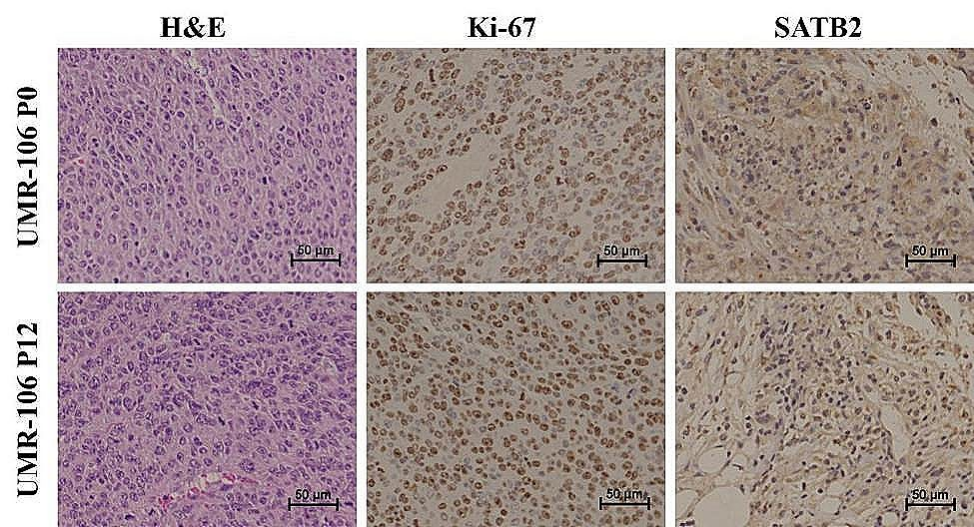
Comparison between the immune-tolerant UMR-106 tumor tissue at generation P12 and the primary UMR-106 tumor tissue at generation P0. (**A**) H&E staining results of generations P0 and P12. (**B**) Ki-67 staining results at generations P0 and P12. (**C**) SATB2 staining outcomes at generations P0 and P12

The H&E staining and immunochemistry results are shown in Fig. 8. Histological sections revealed that large numbers of tumor cells existed in tumor tissues at generations P0 and P12 (Fig. 9A). Large and numerous nuclei and nuclear divisions were observed, suggesting active proliferation of tumor cells [15]. Immunohistochemistry images showed that tumor tissues (P0 and P12 generations) consisted of numerous Ki-67-positive cells (Fig. 9B). This indicated that both generations of tumors possessed exhibited brisk growth and high levels of malignancy. STAB2 (special AT-rich binding protein-2) is a tissue-specific protein with a matrix attachment region and acts as a transcriptional co-factor. It is considered as the marker of osteogenic differentiation for differential diagnosis of osteosarcoma [16]. in-situ-formed tumor cells of tumor tissues at generations P0 and P12 shared the immune-positive signature for STAB2 (Fig. 9C). This finding corroborated the fact that the tumors of generation P12 were osteosarcomas and maintained the characteristics and nature of the tumors at generation P0.

### Construction of orthotopic osteosarcoma model of the distal femur in New Zealand white rabbits

**Fig. 10:**
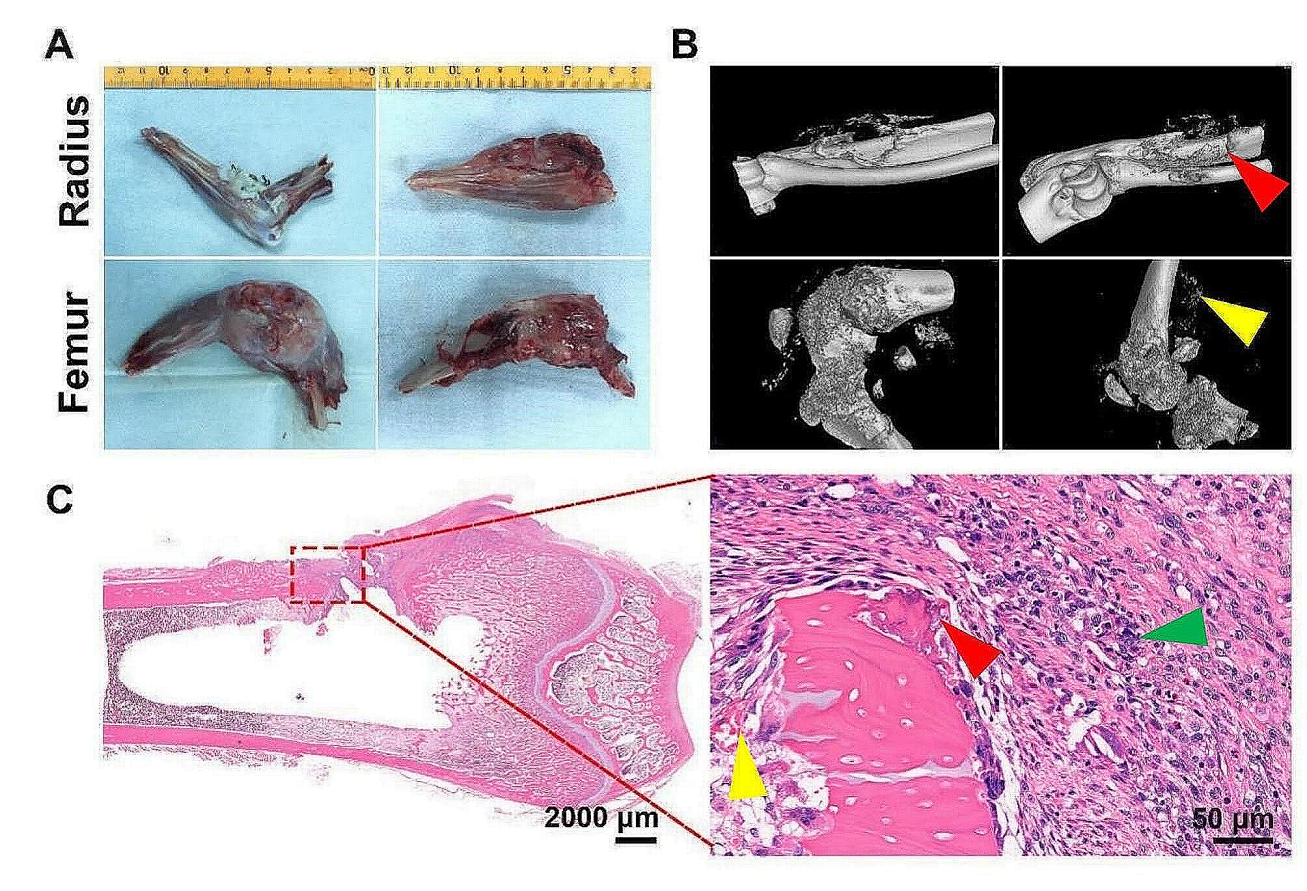
VX2 bone tumors. (**A**) VX2 bone tumors were observed near the pericortical radius and in the femoral condyle. (**B**) 3D micro-CT reconstruction of bone tumors in the distal femur and paracortical radius. (**C**) Orthotopic H&E staining of the VX2 femur tumor

We successfully implanted VX2 tumor tissues in all the studied rabbits with an ideal 100% tumor formation rate (i.e., 6/6) near the pericortical radius or in the bone marrow of New Zealand white rabbits. All tumors were detectable within 12 days of implantation (Fig. 10A). Micro-CT images revealed irregular bone destruction and neoplastic osteolytic lesions, in conjunction with neoplastic osteogenesis, which corresponded to osteosarcoma features (Fig. 10B). As revealed by H&E staining, massive viable tumor cells (green triangle), obvious multiple bone destruction (red triangle), and tumor-like bone matrix (red triangle) were observed (Fig. 10C).

## Discussion

An ideal osteosarcoma model in animals should possess characteristics such as spontaneous tumorigenesis in the bone with pulmonary metastasis and in the host with a intact immune system. However, the currently available osteosarcoma model in mice and rats cannot fulfill the requirements listed above [7]. The choice of grafts and the method used for transplantation are crucial for the successful construction of animal models. It is obvious that orthotopic models, rather than subcutaneous implantations in or on the surface of the bone, justify the fact that tumor grows in the bone, which provides a microenvironment and endogenous growth factors similar to those in the human body via the participation of host stromal cells. In addition, subcutaneous tumor models cannot emulate the process of tumor metastasis to target organs. Fisher et al. first produced the orthotopic model of proximal tibia of athymic mice by inoculating UMR-106-01 cells [17]. A previous study demonstrated that the tumor fragment transplantation method exhibits a higher rate for the successful establishment of tumor models than cell suspension injection [18]. Hildreth et al. reported a mouse model of osteosarcoma wherein tumor fragments were implanted directly into the proximal tibia, with good performance in terms of tumor formation and lung metastasis [19]. Accordingly, we chose the tumor fragment transplantation strategy (Figs. 2 and 3). Considering the smaller body sizes of mice and the participation of the immune system, researchers will likely select rats to construct the animal model of osteosarcoma for the evaluation of surgical methods and the effects of bone-implanted materials [20]. SD rats are extensively used in bone research studies. Moreover, the rat osteosarcoma cell line UMR-106 is usually selected to study the mechanism of osteosarcoma and endocrine and metabolic disorders owing to its strong tumorigenicity and a high lung metastatic capability [20, 21].

UMR-106 cells have high-grade malignancy and active proliferation characteristics; additionally, they present the advantage of interactions between the host microenvironment and tumor cells because this cell line was originally derived from rats with an osteoblastic phenotype [22]. Furthermore, we attempted to construct osteosarcoma models in animals with intact immune systems. To this end, we chose UMR-106 to construct an in-situ osteosarcoma model of the distal femur in SD rats, which showed the same growth pattern as the final stage of osteosarcoma (Fig. 6).

In 2021, Gabrielson et al. successfully construct a syngeneic orthotopic osteosarcoma SD rat model via directly implanting UMR106 OS cell line (originating from a SD rat) with orthotopic tibial tumor implants into 3-week-old male and female rats [23]. Although they obtained immunocompetent tibial osteosarcoma rats, the work does not mention the success rate of the model. According to our experimental results, even with the addition of immunosuppressants, the success rate of such rats’ models is less than 30%. For normal experiments, such a low success rate is costly, labor-intensive, and not conducive to result reproducibility. From a scientific research perspective, this rat model is neither economical nor rigorous. Actually, previous studies demonstrated that immunosuppression is needed to achieve a satisfactory tumor formation rate in rats, even with the use of UMR-106 cells. Cherrier et al. injected tumor cells in the distal femoral medullar cavity to establish a rat osteosarcoma model with the injection of cyclosporine A (10 mg/kg) every day to prevent the immunoreaction until the detection of the tumor. SD rats treated with the immunosuppressive cyclosporine A exhibited extremely high rates of localized tumor formation and pulmonary metastasis [24]. Specifically, we selected an immune-tolerant cell line by gradually decreasing the dose of cyclosporine A. After four rounds of in-vivo selection in immunosuppressed SD rats and six rounds of acclimatization in SD rats with intact immune systems, we obtained immune-tolerant cell lines (UMR-106-P11) and established the orthotopic osteosarcoma model of the distal femur in SD rats, with a 100% tumor formation rate. The morphological characteristics of the new cell line suggested that the junction between cells reduced along with the in vivo selection of immune tolerance (Fig. 8). The reduced connection and loose combination between cells accelerate tumor cell transport from the tumor site into the systemic circulation, and finally the tumor colonizes target organs to form metastasis [25]. Ki-67 protein expression is directly associated with the pathological states of tumor cells and reflects the malignant levels of tumors. An increasing abundance of Ki-67-positive cells indicates the presence of proliferating tumor cell populations with more malignant characteristics (Fig. 9).

This animal model of osteosarcoma met the requirements of primary osteogenic sarcoma in the bone and dissemination to the lung. In aggressive cases or as the tumor progresses, there can be increased osteolytic activity. As the cancer cells proliferate, they can start to invade and resorb the bone more extensively, leading to more pronounced osteolysis. This model exhibited high similarity to human osteosarcoma, with obvious osteolysis (Fig 0.6), and preserved the histological characteristics of primary osteosarcoma (Fig. 7) after a few rounds of screening. Because of the intact immune systems, it allows researchers further explore the immune protection and the role of immune system in tumorigenesis and metastasis.

New Zealand White rabbits have larger bones than rat, which can provide more convenience for the treatment of osteosarcoma, especially in surgical operations and bone-implanted materials research [26]. Researchers commonly select the VX2 carcinoma to construct rabbit model to simulate the process of bone tumor biology, including tumor proliferation, invasion, and metastasis, mainly because VX2 is an anaplastic squamous cell carcinoma [27], with characteristics such as rapid growth, ideal tumor growth rate, and good comparability, similar to those of human malignant bone tumors [27, 28]. In a previous study, Melancon et al. discussed the antitumor effects of irreversible electroporation, used alone or in combination with doxorubicin–loaded superparamagnetic iron oxide nanoparticles, in a VX2 rabbit tibial tumor model [29]. Moreover, the rabbit model is also useful for the evaluation of new techniques in oncologic imaging [30, 31]. In this study, the histological and imaging characteristics of the osteosarcoma model of New Zealand white rabbits presented herein are similar to those reported in previous studies (Fig. 10). These findings presented the natural course of the rabbit after VX2 carcinoma implantations into the bone (tibia), including lung metastasis, pathologic fractures, and the changes in serum marker levels [32]. The implantation of VX2 tumor fragments into the rabbit bones is an effective method to obtain an osteosarcoma model in large animals for future evaluation of surgical operations, image diagnostics, and bone materials.

## Conclusions

In our study, the orthotopic animal models of osteosarcoma constructed in SD rats and New Zealand white rabbits demonstrated the histological and imaging characteristics of osteosarcoma, with similar features to those of clinical osteosarcoma. These included bone destruction, neoplastic osteogenic calcification, and lung metastasis. Moreover, the immune-tolerant UMR-106 cell line with strong metastatic ability was isolated from the tumors formed in SD rats and exhibited biological behavior that was close to the clinical course of osteosarcoma. These animal models of osteosarcoma are expected to have excellent prospects for potential use in biological and pathogenetic studies of osteosarcoma and are envisaged to be valuable to study various tumor-stroma communications, particularly the function of immune cells surrounding the tumor cells. Especially, the size of the animals allows a better handling and evaluating the surgical treatment and bone materials for clinical translation.

## Data Availability

All raw data from characterization are available from the corresponding author upon request.
